# Get Vigorous with Physical Exercise and Improve Your Well-Being at Work!

**DOI:** 10.3390/ijerph17176384

**Published:** 2020-09-02

**Authors:** Ester Gil-Beltrán, Isabella Meneghel, Susana Llorens, Marisa Salanova

**Affiliations:** 1 WANT Research Team, Universitat Jaume I, Av. Vicent Sos Baynat, s/n Castellón de la Plana, 12071 Castellón, Spain; llorgum@uji.es (S.L.); salanova@uji.es (M.S.); 2 Àrea de Psicologia i Salut Mental, Universitat Internacional de Catalunya, San Cugat del Vallés, 08195 Barcelona, Spain; imeneghel@uic.es

**Keywords:** physical exercise, well-being, vigor, job satisfaction, positive affect, stress, healthy organizations

## Abstract

The aim of this study is to investigate whether people who exercise regularly have higher levels of psychological well-being at work. Doing physical exercise is a habit that not only has consequences for physical and mental health, but it can also have positive consequences for organizations because physical exercise makes it easier for the employee to recover from physical, mental, and emotional effort during the workday, thus showing higher levels of engagement the next day. Through the analysis of structural equation models in a sample of 485 workers from different Spanish and Latin American companies, this study shows that subjects who exercise more have higher levels of vigor in physical exercise, which is positively related to high levels of well-being at work. This means that organizations that promote activities related to physical exercise among their employees are building a process of resource recovery, which, through the vigor of these activities, makes workers feel less stressed and more satisfied, thus experiencing greater well-being at work. Therefore, at a practical level, these results suggest that the practice of physical exercise is a tool for organizations that want to promote their employees’ psychological well-being.

## 1. Introduction

In the past century, the world has evolved considerably at a scientific and technological level, especially in developed countries where this progression is more evident. What human beings did for millions of years was dependent exclusively on their physical capacity, but today machines do this work, factories have been automated, transport has been mechanized, and even for housework, countless household appliances have been invented that are increasingly autonomous. All these results have caused the physical activity that was carried out in the past to be considerably reduced [1].

According to the latest data, the World Health Organization (WHO) [2] estimates that at least 23% of the adult population and 81% of adolescents worldwide have physical activity levels that are below those necessary to maintain health and control their body weight. This lack of activity can be associated with non-communicable diseases, depression and anxiety problems [3], or lack of vigor [4].

A possible solution to this sedentary lifestyle and its negative consequences would be the practice of Physical Exercise (PE). Acevedo [5] conceptualizes it as “a structured form of physical activity, with the specific objective of improving or maintaining physical health or fitness” (p. 4). In addition, he shows that the recurrent and sustained practice of PE can lead to physical benefits at the cardiorespiratory, muscular, and bone levels, as well as reducing the risk of non-communicable diseases.

However, would PE have other benefits? The answer is yes because previous studies have shown that it can also be valuable at a physiological level: PE increases levels of endocannabinoids [6,7], endorphins [8], serotonin [9], and dopamine [10,11]. These neurotransmitters are responsible for pain reduction, emotion regulation, pleasure [12], and stress reduction [13]. In addition, the practice of PE would also be beneficial for improving psychological well-being: PE helps to provide mental distraction from workday demands [14], and both the feeling of mastery and the increase in self-efficacy when performing PE can facilitate recovery from stress levels [15,16].

Therefore, in the long term, the practice of PE would also result in benefits for organizations. In fact, healthy employees who feel good, are resilient in the face of stressful situations, and experience positive emotions have better performance in terms of economic or quality results [17,18]. Along these lines, there is evidence that organizations that are considered healthy adopt PE intervention mechanisms aimed at increasing positive emotional states and, collaterally, performance [19].

Even though there is evidence about PE’s relevance for people and, by extension, for organizations, research on the impact of PE on employee well-being is extremely scarce. For all these reasons, the objective of this study is to contribute to expanding this research by investigating the relationship between the amount of time of PE and well-being at work, understood as job satisfaction, the presence of positive affect in the work environment and absence of stress. Furthermore, another objective is to investigate the modulating effect of vigor in PE on this relationship.

### 1.1. Physical Exercise

According to Sonnentag [20], PE can be understood as a recovery activity and, therefore, a promoter of psychological well-being. Recovery after a workday is a process that would allow the psychological systems, which have been subjected to stress during the workday, to return to their pre-stress levels [21]. Recovery is theoretically based on Conservation of Resources Theory [22,23] and the Effort-Recovery Model [21]. These theories propose that people tend to recover these resources that are depleted during stressful situations because they have the motivation to conserve, promote, and protect their resources [22,23]. At the same time, people tend to move away from sources of stress with the same intention of recovering and returning to the levels they had before the stressful situation [21].

Performing PE makes us obtain energy resources that can be categorized into three types: (1) physical resources, such as better aerobic or cardiorespiratory capacity or greater muscle power; (2) cognitive resources because, according to Yeung [14], PE can be a good mental distractor from job demands; more importantly for our study—(3) emotional resources, because research has shown that performing PE activates the left prefrontal cortex and, as explained above, releases neurotransmitters that are related to pleasure, motivation, and regulation of emotions [24].

As noted above, PE, in addition to physical benefits, produces benefits on an emotional and psychological level. In their study, Nägel, Sonnentag and Kühnel [19] show that on the days when employees do PE after their workday, they perceive an improvement in their positive affect, understood as the presence of positive emotional states, and in the serenity they experience before going to bed. Moreover, these benefits at the emotional level extend to the organizational level because there is ample evidence that positive affective states are important antecedents of good work results and success [25,26,27,28]. Along these lines, recently, non-sedentary people were shown to be more empathetic and more absorbed in their work tasks [29].

### 1.2. Well-Being at Work

The concept of well-being can be understood from two perspectives: eudaimonic, and hedonic well-being. Eudaimonic well-being, also called psychological well-being, establishes that well-being lies in carrying out activities that are consistent with one’s vital values [30]. Hedonic well-being, also called subjective well-being, has the life goal of accumulating experiences of pleasure [31], and it consists of two elements: on the one hand, the affective balance, that is, the difference between the positive and negative emotions experienced; on the other hand, life satisfaction, which is an overall judgment of one’s life [32].

The present study refers to three indicators of subjective well-being at work resulting from PE. The variables taken into consideration are: (1) job satisfaction, understood as a pleasant or positive state that results from the positive evaluation of work or work experiences; (2) positive affect, understood as the presence of positive emotional states in the work environment; (3) stress (as an indicator of absence of well-being), understood as the employee’s perception of having worries and pressures that negatively alter his/her mood. According to the Conservation of Resources Theory [22] and the Effort-Recovery Model [21], mentioned above, the practice of PE is expected to have a positive impact on the indicators of well-being at work. That is, the greater the amount of PE carried out, the higher the levels of job satisfaction and positive affect, and the lesser the level of stress.

### 1.3. Vigor in Physical Exercise

With the aim of enhancing the effects of PE on work well-being, this study proposes that vigor in physical exercise can act as a modulator. Vigor, according to Schaufeli, Salanova, González-Romá and Bakker [33], is one of the dimensions of engagement, and it is characterized by high levels of energy and the desire to invest effort in the activity being performed, even when difficulties appear along the way. Therefore, vigor can be considered a motivational measure. In this study, transferring this definition to the vigor in PE, it can be understood as the motivation that drives the performance of PE and the will to persistently invest effort in it.

This definition proposes that vigor when performing PE enhances the positive effects of PE on work well-being. If people feel this vigor when doing PE, the acquisition, retention and/or protection of all the energy resources that are obtained from it, and that lead to positive emotions, are fostered. Therefore, vigor is a key element in PE that helps us to continue to obtain the resources PE provides and increases its positive effects on well-being.

### 1.4. Hypotheses of the Study

The objective of the present study is to investigate the relationship between PE practice and work well-being, emphasizing the modulating effect of vigor in PE on this relationship. Thus, the effect of vigor is expected to act as an enhancer of the relationship proposed. Therefore, PE would have a direct positive effect on the indicators of work well-being. Experiencing the sensation of energy and the desire to invest effort in PE, that is, high levels of vigor, would lead to higher levels of work well-being.

**Figure 1:**
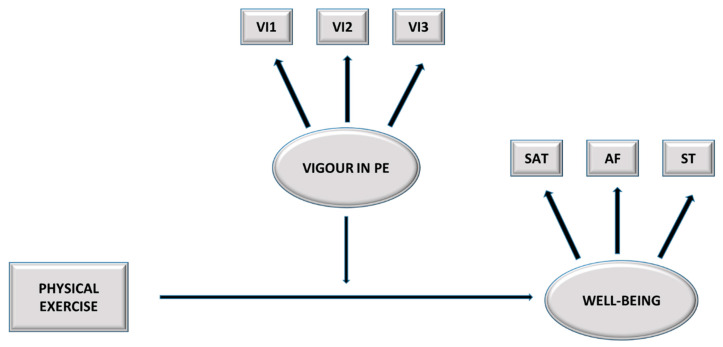
Hypothesized model. PE = Physical Exercise; VI1 = Item 1; VI2 = Item 2; VI3 = Item 3; SAT = Job satisfaction, AF = Positive affect, ST = Stress.

Specifically, the study hypotheses are as follows (see Figure 1):

**Hypothesis** **1.**  *A positive and significant relationship is expected between PE and well-being at work. That is, the more PE, the higher the levels of well-being (higher job satisfaction and positive affect and lower stress).*

**Hypothesis** **2.**  *Vigor is expected to modulate the relationship between PE and well-being at work, so that when vigor in PE is higher, the relationship between PE and well-being will be more intense.*

## 2. Materials and Methods

### 2.1. Participants and Protocol

The total sample was made up of 485 employees who do PE at least 90 minutes a week, which is the cut-off point proposed by WHO to distinguish between sedentary and active people [34]. In total, 52% of the employees are women, the average age is 39.20 years (minimum = 19; maximum = 64, SD = 8.8), and 77% had an indefinite contract.

Regarding the procedure, the participants completed the questionnaire in its online format, with the prior consent of the companies’ management. Likewise, and ensuring the confidentiality of the data at all times, when starting the questionnaire, they were asked for their consent for these data to be processed only for scientific and non-profit purposes, according to the Data Protection Law. Finally, in order to complete the questionnaire, a link was provided.

### 2.2. Measures

Relying on evidence of the validity of using single-item scales when evaluating variables such as Satisfaction or Engagement [35,36], and with the aim of reducing response time, which is highly demanded by companies and the subjects themselves, some of the scales that make up the questionnaire consist of a single item. The variables and questionnaires used for the study are described below:

- Physical exercise. It was evaluated as the amount of time the participants spent carrying out physical exercise during the week. For each sport they practiced, they were asked to indicate how long the session/s was/were. For the study, it was taken into account the sum of the minutes of all the exercises performed during the week (minimum = 90 min; maximum = 1560 min; mean = 266.50 min; SD = 179.27).
- Vigor in physical exercise. It was evaluated using the three items corresponding to the vigor dimension of the Utrecht Work Engagement Scale (UWES-9) [37], but adapted to PE (“When I do physical exercise, I feel full of energy”; “When I wake up in the morning, I feel like going to do physical exercise”; “Due to physical exercise, I feel strong and energetic”). It was measured with a Likert-type scale ranging from 0 (never) to 6 (always).
- Job satisfaction. It was evaluated using a scale of faces [38]; on a single item, they were asked to indicate the face that best expressed their degree of satisfaction with their work (0 = sad face, very dissatisfied and 6 = face happy, very satisfied).
- Positive affect. It was evaluated using a 7-point visual analog scale [39], where a single item asked them to indicate the face that best expressed how they had felt at the level of emotional well-being during the past year at work (0 = sad face and 6 = happy face).
- Stress. It was evaluated using the one item corresponding to the stress dimension of the General Health Questionnaire (GHQ) [40] adapted to the work context (“In my work, my worries have taken my sleep away”). It was measured with a Likert-type scale ranging from 0 (much less than usual) to 6 (much more than usual).

### 2.3. Data Analysis

First, internal consistency analyses (Cronbach’s alpha), descriptive analyses (means, standard deviations), and internal correlations of the variables considered in the study were performed using the IBM SPSS Statistics 24.0 statistical package (IBM, Armonk, NY, USA). Second, the single-factor Harman test [41] was performed using the AMOS 24.0 statistical package (IBM, Armonk, NY, USA) to check for common variance bias.

Hierarchical regression analyses were then performed to assess modulation effects [42], using each of the three indicators of well-being at work as a dependent variable. To test modulation, the procedure pretends to enter the independent and modulating variables into the equations in three successive steps [43]. In the first step, the amount of time the participants spent carrying out physical exercise during the week (independent variable) was introduced. In the second step, vigor with the PE (modulator) was introduced. Finally, in the third step, the interaction between the independent and the modulator variables was included.

## 3. Results

### 3.1. Descriptive Analyses and Harman’s Test

Table 1 shows the means, standard deviations, and intercorrelations between the study variables. The results show that the scale of vigor in PE (α = 0.75) meets the reliability criterion proposed by the scientific research [44]; the rest of the variables had one item and, therefore, reliability cannot be measured. Correlation analyses show that the variables are positively related to each other, except for the amount of time spent doing PE, which only correlates with vigor in PE (see Table 1).

Second, the results of the Harman single factor test revealed a poor fit of the data, χ^2^ (9) = 427.42, Root Mean Square Error of Approximation (RMSEA) = 0.31, Comparative Fit Index (CFI) = 0.55, Tucker–Lewis Index (TLI) = −0.06, Incremental Fit Index (IFI) = 0.55. Furthermore, following the recommendations of Podsakoff, Mackenzie, and Podsakoff [45], in order to minimize the impact of common method variance bias, the questionnaire had different headings to differentiate its different parts, as well as different response scales. Therefore, it can be considered that this bias does not affect the study data, and so the variance in the variables can be attributed to the evaluated constructs, and not to the evaluation method.

### 3.2. Hierarchical Regression Analysis.

The results of the regression analyses show that there is a not significant relationship between the amount of time spent in PE with well-being (β = 0.06, *p* = 0.16). Thus, steps 2 and 3 of the modulation analyses were not performed. These results indicate that neither H1 nor H2 is confirmed.

### 3.3. Additional Analyses

As the results of the evaluation of the modulation effects showed that vigor in PE did not modulate the relationship between PE and work well-being, but there was a relationship between vigor in PE and well-being, additional analyses were performed. The analyses carried out were aimed at verifying whether vigor in PE acts as a mediator, rather than as a modulator, in the relationship between PE and well-being at work. For this purpose, Structural Equation Modeling (SEM) was carried out.

The SEM was performed using the AMOS 25.0 program, testing two models [46]: (M1) the physical exercise model, which proposes that vigor in PE fully mediates the relationship between PE and well-being; the alternative model (M2), which proposes that well-being mediates the relationship between PE and vigor in PE. Furthermore, to test mediation, the MacKinnon et al. test [47] was performed. To do this, three steps were taken to estimate: (1) the unstandardized weight of PE on vigor in PE (α), (2) the unstandardized weight of vigor in PE on well-being (β), and (3) the product of the previous effects (αβ). To determine whether mediation was full or partial, the direct effect from the independent variable to the dependent variable (τ) was calculated, which must not be significant in order for full mediation to occur.

Maximum likelihood estimation methods were used with the calculation of the absolute and relative goodness of fit indices [48]: the Chi-square index (*p* > 0.05), relative chi-square index (chi-square/gl; up to 5.0), Root Mean Square Error of Approximation (RMSEA), as well as the Comparative Fit Index (CFI), the Tucker–Lewis Index (TLI), and the Incremental Fit Index (IFI). Values below 0.08 and higher than 0.90 indicate a good fit for RMSEA [49] and for the rest of the indices [50], respectively. Furthermore, the Akaike Information Criterion (AIC; [51]) was calculated to compare non-nested competitive models (i.e., M1 versus M2); the lower the AIC levels, the better the fit.

#### Model Fit: Structural Equation Models

Table 2 shows the SEM results for the model tests. The variables of vigor and well-being were latent variables, composed of three indicators each, whereas PE variable was observable.

The SEM results indicate that the hypothesized M1, where vigor in PE mediates the relationship between PE and job satisfaction and positive affect, χ^2^ (2) = 23.36, RMSEA = 0.04, CFI = 0.99, TLI = 0.98, IFI = 0.99, AIC = 67.36, fits better than M2, the alternative model, where well-being mediates the relationship between PE and vigor in PE χ^2^ (2) = 55.96, RMSEA = 0.80, CFI = 0.95, TLI = 0.90, IFI = 0.96, AIC = 99.96, because its AIC (67.36) is lower.

Furthermore, following the MacKinnon et al. product of coefficients method [47], the hypothesized model, meets all the requirements for a full mediation effect of vigor in PE on the dependent variable. The results were (1) the amount of time spent on PE is positively and significantly related to vigor in PE, α = 0.01, *p* < 0.001; (2) vigor in PE is significantly and positively related to well-being, β = 0.23, *p* < 0.001; (3) the mediation effect is positive and statistically significant, αβ = 0.03, *p* < 0.01. These results show that vigor in PE mediates the relationship between the frequency of PE and well-being at work. This mediation is full because the direct relationship between the independent and the dependent variable is not significant: amount of time of PE and well-being τ = 0.00, *p* = 0.86.

**Figure 2:**
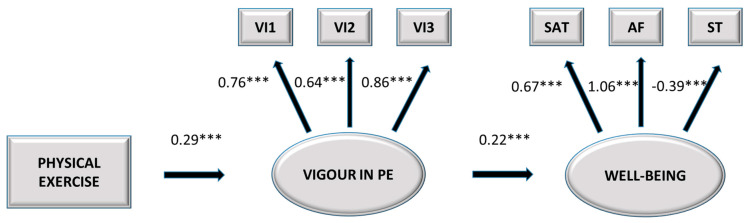
Final model. VI1 = Item 1; VI2 = Item 2; VI3 = Item 3; SAT = Job satisfaction, AF = Positive affect, ST = Stress. *** *p* < 0.001.

The final model is depicted in Figure 2.

## 4. Discussion

The objective of the present study was to investigate the relationship between PE practice and employee well-being, emphasizing the modulating effect of vigor in PE in this relationship. The results showed that this modulation was not significant. In fact, there was no direct relationship between the PE performed and the workplace well-being variables. This leads us to reject both Hypothesis 1 and Hypothesis 2, and to consider the possible mediator effect of vigor in PE, which would mean that the way the PE is psychologically experienced is important with respect to PE motivation (i.e., high levels of energy, feeling strong and vigorous doing PE, and feeling like doing it) in achieving higher level of well-being. This idea was tested by SEM analysis, and the results showed that, indeed, there was a full mediation by vigor in PE in the relationship between the PE carried out and well-being at work. These results suggest that people who do PE and feel vigorous/motivated about it experience higher levels of workplace well-being, lower level of stress, and greater satisfaction with their work, as well as positive emotions.

These results could be explained by what Catalino et al. [52] call “prioritization of the positive”. This concept is based on the formula of happiness by Lyubomirsky et al. [27]. This formula states that happiness is made up of genetics, circumstances, and intentional activities. Based on this latter element, Catalino et al. [52] established that people who organize their daily lives looking for positivity, that is, taking into account their potential happiness, have more positive emotions and higher levels of satisfaction with life. In our study, vigor in PE means high energy levels and the desire to invest effort in the activity carried out, even when difficulties appear along the way. Thus, there is an intention and motivation for a healthy activity.

Another possible explanation would be that PE promotes vigor through a motivational process that meets the basic need for autonomy, relationship, and competence, as postulated in the Self-Determination theory [53]. For example, regular physical exercise encourages improvement, thus increasing competition, whereas determining what your exercise goals are satisfies autonomy needs, and sharing moments of physical exercise with others satisfies the need to belong, respectively. Therefore, people who feel vigorous have a feeling of energetic and affective connection with PE.

Both explanations would lead us to positive affects, either by searching for them through PE (prioritization of the positive) or as a result of satisfying basic needs when doing PE, and positive affects are important antecedents of work-related outcomes and success [25,27,28].

In short, the results indicate that performing PE (measured through its duration) is a way of encouraging workers to be satisfied and emotionally positive in their work, as long as they experience vigor when doing it.

### 4.1. Theoretical and Practical Implications

From a theoretical point of view, this study expands the investigation of the effects of PE on the level of psychological well-being at work. Specifically, the importance of PE is evident, but there is a need for motivation, a perceived vigor, so that these optimal levels of well-being at work can occur.

From a practical point of view, taking into account that doing PE continuously is a source of psychological well-being at work, it would be a good strategy for organizations to encourage their employees to do PE. However, it is not enough to simply do PE, but important that employees feel vigorous and motivated to do it! So, wellness practices from organizations should contemplate PE programs for organized activities or set up facilities within the organization itself, so that workers do not have to travel to do sports, or they can contribute by providing a discount on part of the registration fee in sports centers. These practices to enhance PE should be attractive for employees in order that they feel motivated and vigorous for doing it, and then they could improve their levels of employee well-being.

### 4.2. Study Limitations and Future Research

The present study has various limitations. In the first place, it was a convenience sample that was used, which compromises the generalization of the results obtained. However, the data have the strength of including workers from different labor contexts and countries. The second limitation is that it is a cross-sectional study. Finally, another limitation is that given that vigor is a motivational measure which reflects a cognitive/emotional construct, some potential multicollinearity issues with the data (especially job satisfaction and positive affect) can be found.

Future studies should include longitudinal designs to test the effect of PE and vigor in PE on well-being at work. These studies could also show evidence of the effects of PE as a stress recovery activity and promoter of positive emotions at work, and they could also incorporate job performance into the equation.

It would also be interesting to test the possible explanations for the mediation of vigor in PE, collecting data on the subjects’ “prioritization of the positive” and their motivations for performing PE.

Finally, in this study we have seen that there is not a significant effect of PE on well-being indicators; however, we have also seen that the more PE, the more vigor in PE which will, in turn, provide higher levels of job satisfaction and positive affect. Future experimental and longitudinal studies may deepen the study of the minimum frequency of PE that provides enough vigor in the PE so that employees feel higher levels of job satisfaction and positive affect.

## 5. Conclusions

The results of this study show that there is no direct relationship between the PE performed and well-being at work, as pointed out in the introduction. What this study shows is that in order to decrease stress level and improve job satisfaction levels and make work emotions more positive, it is not enough to just carry out PE regularly, but there must also be a motivation to experience vigor when doing it.

## Figures and Tables

**Table 1:** Descriptive statistics and correlations between the study variables.

	Variables	M	SD	1	2	3	4	5
1	Amount time PE	266.50	179.27	-				
2	Vigor PE	4.21	1.09	0.28 **	-			
3	Job satisfaction	4.46	1.19	0.04	0.07	-		
4	Positive affect	4.11	1.40	0.07	0.18 **	0.71 **	-	
5	Stress	2.81	1.88	−0.08	−0.12 **	−0.26 **	−0.42 **	-

Note. The correlation is significant at the level of ** *p* < 0.01 (bilateral). PE = Physical Exercise; M = Means; SD = Standard Deviations.

**Table 2:** Fit indices for structural equation models.

Model	χ^2^	gl	χ^2^/gl	RMSEA	CFI	IFI	TLI	AIC
M1	23.36	13	1.80	0.04	0.99	0.99	0.98	67.36
M2	55.96	13	4.30	0.08	0.95	0.96	0.90	99.96

Notes: χ^2^ = Chi-square; gl = degrees of freedom; χ^2^/gl = relative Chi-square; RMSEA = Root Mean Square Error of Approximation; CFI = Comparative Fit Index; TLI = Tucker–Lewis Index; IFI = Incremental Fit Index; AIC = Akaike Information Criterion. M1 = Physical exercise model; M2 = Alternative model.

## References

[1] Jackson A.W., Morrow J.R., Hill D.W., Dishman R.K. (2006). Physical Activity for Health and Fitness.

[2] World Health Organization Physical Activity. https://www.who.int/news-room/facts-in-pictures/detail/physical-activity.

[3] Fox K.R. (1999). The Influence of Physical Activity on Mental Well-Being. Public Health Nutr..

[4] Lee R.E., Goldberg J.H., Sallis J.F., Hickmann S.A., Castro C.M., Chen A.H. (2001). A Prospective Analysis of the Relationship between Walking and Mood in Sedentary Ethnic Minority Women. Women Health.

[5] Acevedo E.O., Acevedo E.O. (2012). Exercise Psychology: Understanding the Mental Health Benefits of Physical Activity and the Public. The Oxford Handbook of Exercise Psychology.

[6] Marco E.M., García-Gutiérrez M.S., Bermúdez-Silva F.J., Moreira F.A., Guimarães F., Manzanares J., Viveros M.P. (2011). Endocannabinoid System and Psychiatry: In Search of a Neurobiological Basis for Detrimental and Potential Therapeutic Effects. Front. Behav. Neurosci..

[7] Raichlen D.A., Foster A.D., Seillier A., Giuffrida A., Gerdeman G.L. (2013). Exercise-Induced Endocannabinoid Signaling Is Modulated by Intensity. Eur. J. Appl. Physiol..

[8] Boecker H., Spilker M.E., Henriksen G., Koppenhoefer M., Wagner K.J., Valet M., Berthele A., Tolle T.R. (2008). The Runner’s High: Opioidergic Mechanisms in the Human Brain. Cereb. Cortex.

[9] Wipfli B., Landers D., Nagoshi C., Ringenbach S. (2011). An Examination of Serotonin and Psychological Variables in the Relationship between Exercise and Mental Health. Scand. J. Med. Sci. Sports.

[10] Berse T., Rolfes K., Barenberg J., Dutke S., Kuhlenbäumer G., Völker K., Winter B., Wittig M., Knecht S. (2015). Acute Physical Exercise Improves Shifting in Adolescents at School: Evidence for a Dopaminergic Contribution. Front. Behav. Neurosci..

[11] Heyman E., Gamelin F.X., Goekint M., Piscitelli F., Roelands B., Leclair E., Di Marzo V., Meeusen R. (2012). Intense Exercise Increases Circulating Endocannabinoid and BDNF Levels in Humans-Possible Implications for Reward and Depression. Psychoneuroendocrinology.

[12] Heijnen S., Hommel B., Kibele A., Colzato L.S. (2016). Neuromodulation of Aerobic Exercise—A Review. Front. Psychol..

[13] Suzuki W., Fitzpatrick B. (2015). Cerebro Activo, Vida Feliz.

[14] Yeung R.R. (1996). The Acute Effects of Exercise on Mood State. J. Psychosom. Res..

[15] Demerouti E., Bakker A.B., Geurts S.A.E., Taris T.W. (2009). Daily Recovery from Work-Related Effort during Non-Work Time. Res. Occup. Stress Well Being.

[16] Sonnentag S., Jelden S. (2009). Job Stressors and the Pursuit of Sport Activities: A Day-Level Perspective. J. Occup. Health Psychol..

[17] Salanova M., Llorens S., Cifre E., Martínez I.M. (2012). We Need a Hero! Toward a Validation of the Healthy and Resilient Organization (HERO) Model. Group Organ. Manag..

[18] Salanova M., Llorens S., Martínez I.M. (2019). Organizaciones Saludables. Una Mirada Desde La Psicología Positiva.

[19] Nägel I.J., Sonnentag S., Kühnel J. (2015). Motives Matter: A Diary Study on the Relationship between Job Stressors and Exercise after Work. Int. J. Stress Manag..

[20] Sonnentag S. (2001). Work, Recovery Activities, and Individual Well-Being: A Diary Study. J. Occup. Health Psychol..

[21] Meijman T.F., Mulder G., Drenth P.J.D., Thierry H. (1998). Psychological Aspects of Workload. Handbook of Work and Organizational Psychology. Volume 2: Work Psychology.

[22] Hobfoll S.E. (1998). Stress, Culture, and Community: The Psychology and Philosophy of Stress.

[23] Hobfoll S.E. (2001). The Influence of Culture, Community, and the Nested-Self in the Stress Process: Advancing Conservation of Resources Theory. Appl. Psychol..

[24] Basso J.C., Suzuki W.A. (2017). The Effects of Acute Exercise on Mood, Cognition, Neurophysiology, and Neurochemical Pathways: A Review. Brain Plast..

[25] Erez A., Isen A.M. (2002). The Influence of Positive Affect on the Components of Expectancy Motivation. J. Appl. Psychol..

[26] Ilies R., Judge T.A. (2005). Goal Regulation across Time: The Effects of Feedback and Affect. J. Appl. Psychol..

[27] Lyubomirsky S., King L., Diener E. (2005). The Benefits of Frequent Positive Affect: Does Happiness Lead to Success?. Psychol. Bull..

[28] Tsai W.C., Chen C.C., Liu H.L. (2007). Test of a Model Linking Employee Positive Moods and Task Performance. J. Appl. Psychol..

[29] Gil-Beltrán E., Llorens S., Salanova M. (2020). Employees’ Physical Exercise, Resources, Engagement, and Performance: A Cross-sectional Study from HERO Model. J. Work Organ. Psychol..

[30] Waterman A.S. (1993). Two Conceptions of Happiness: Contrasts of Personal Expressiveness (Eudaimonia) and Hedonic Enjoyment. J. Personal. Soc. Psychol..

[31] Vázquez C., Hervás G., Rahona J.J., Gómez D. (2009). Bienestar Psicológico y Salud: Aportaciones Desde La Psicología Positiva. Anuario de Psicología Clínica y de la Salud.

[32] Lucas R.E., Diener E., Suh E. (1996). Discriminant Validity of Well-Being Measures. J. Personal. Soc. Psychol..

[33] Schaufeli W., Salanova M., González-romá V., Bakker A. (2002). The Measurement of Engagement and Burnout: A Two Sample Confirmatory Factor Analytic Approach. J. Happiness Stud..

[34] World Health Organization OMS|Recomendaciones Mundiales Sobre la Actividad Física para la Salud. https://www.who.int/dietphysicalactivity/factsheet_recommendations/es/.

[35] Nagy M.S. (2002). Using a Single-Item Approach to Measure Facet Job Satisfaction. J. Occup. Organ. Psychol..

[36] Schaufeli W.B., Shimazu A., Hakanen J., Salanova M., De Witte H. (2019). An Ultra-Short Measure for Work Engagement The UWES-3 Validation Across Five Countries. Eur. J. Psychol. Assess..

[37] Schaufeli W.B., Bakker A.B., Salanova M. (2006). The Measurement of Work Engagement the Measurement of Work Engagement with a Short Questionnaire A Cross-National Study. Educ. Psychol. Meas..

[38] Kunin T. (1955). The Construction of a New Type of Attitude Measure. Pers. Psychol..

[39] Fernández-Castro J., Martínez-Zaragoza F., Rovira T., Edo S., Solanes-Puchol Á., Martín-del-Río B., García-Sierra R., Benavides-Gil G., Doval E. (2017). How Does Emotional Exhaustion Influence Work Stress? Relationships between Stressor Appraisals, Hedonic Tone, and Fatigue in Nurses’ Daily Tasks: A Longitudinal Cohort Study. Int. J. Nurs. Stud..

[40] Sánchez López M.P., Dresch V. (2008). The 12-Item General Health Questionnaire (GHQ-12): Reliability, External Validity and Factor Structure in the Spanish Population. Psicothema.

[41] Podsakoff P.M., MacKenzie S.B., Lee J.Y., Podsakoff N.P. (2003). Common Method Biases in Behavioral Research: A Critical Review of the Literature and Recommended Remedies. J. Appl. Psychol..

[42] Cohen J., Cohen P. (1983). Applied Multiple Regression/Correlation Analysis for Behavioral Sciences.

[43] Aiken L.S., West S.G., Reno R.R. (1991). Multiple Regression: Testing and Interpreting Interactions.

[44] Nunnally J.C., Bernstein I.H. (1994). The Assessment of Reliability. Psychometric Theory.

[45] Podsakoff P.M., MacKenzie S.B., Podsakoff N.P. (2012). Sources of Method Bias in Social Science Research and Recommendations on How to Control It. Annu. Rev. Psychol..

[46] James L.R., Mulaik S.A., Brett J.M. (2006). A Tale of Two Methods. Organ. Res. Methods.

[47] MacKinnon D.P., Lockwood C.M., Hoffman J.M., West S.G., Sheets V. (2002). A Comparison of Methods to Test Mediation and Other Intervening Variable Effects. Psychol. Methods.

[48] Marsh H.W., Balla J.R., Hau K.T., Marcoulides G.A., Schumacker R.E. (1996). An Evaluation of Incremental Fit Indices: A Clarification of Mathematical an Empirical Properties. Advanced Structural Equation Modeling, Issues and Techniques.

[49] Browne M.W., Cudeck R. (1992). Alternative Ways of Assessing Model Fit. Sociol. Methods Res..

[50] Hornung S., Glaser J. (2010). Employee Responses to Relational Fulfilment and Work-Life Benefits: A Social Exchange Study in the German Public Administration. Int. J. Manpow..

[51] Akaike H. (1987). Factor Analysis and AIC. Psychometrika.

[52] Catalino L.I., Algoe S.B., Fredrickson B.L. (2014). Prioritizing Positivity: An Effective Approach to Pursuing Happiness?. Emotion.

[53] Ryan R., Deci E. (2000). Self-Determination Theory and the Facilitation of Intrinsic Motivation, Social Development, and Well-Being. Am. Psychol..

